# How Habitat Change and Rainfall Affect Dung Beetle Diversity in Caatinga, a Brazilian Semi-Arid Ecosystem

**DOI:** 10.1673/031.011.11401

**Published:** 2011-09-06

**Authors:** Carolina Nunes Liberal, Ângela Maria Isidro de Farias, Marcos Vinicius Meiado, Bruno K. C. Filgueiras, Luciana Iannuzzi

**Affiliations:** ^1^ Programa de Pós-Graduação em Biologia Animal, Universidade Federal de Pernambuco, Av. Professor Moraes Rego s/n, Cidade Universitária, 50670-901, Recife, Pernambuco, Brazil; ^2^ Departamento de Zoologia, Universidade Federal de Pernambuco, Av. Professor Moraes Rego s/n, Cidade Universitária, 50670-901, Recife, Pernambuco, Brasil; ^3^ Programa de Pós-Graduação em Biologia Vegetal, Universidade Federal de Pernambuco, Av. Professor Moraes Rego s/n, Cidade Universitária, 50670-901, Recife, Pernambuco, Brazil

**Keywords:** dry season, human impact, Scarabaeinae

## Abstract

The aim of the present study was to evaluate how dung beetle communities respond to both environment and rainfall in the Caatinga, a semi-arid ecosystem in northeastern Brazil. The communities were sampled monthly from May 2006 to April 2007 using pitfall traps baited with human feces in two environments denominated “land use area” and “undisturbed area.” Abundance and species richness were compared between the two environments and two seasons (dry and wet season) using a generalized linear model with a Poisson error distribution. Diversity was compared between the two environments (land use area and undisturbed area) and seasons (dry and wet) using the Two-Way ANOVA test. Non-metric multidimensional scaling was performed on the resemblance matrix of Bray-Curtis distances (with 1000 random restarts) to determine whether disturbance affected the abundance and species composition of the dung beetle communities. Spearman's correlation coefficient was used to determine whether rainfall was correlated with abundance and species richness. A total of 1097 specimens belonging to 13 species were collected. The most abundant and frequent species was *Dichotomius geminatus* Arrow (Coleoptera: Scarabaeidae). The environment exerted an influence over abundance. Abundance and diversity were affected by season, with an increase in abundance at the beginning of the wet season. The correlation coefficient values were high and significant for abundance and species richness, which were both correlated to rainfall. In conclusion, the restriction of species to some environments demonstrates the need to preserve these areas in order to avoid possible local extinction. Therefore, in extremely seasonable environments, such as the Caatinga, seasonal variation strongly affects dung beetle communities.

## Introduction

Tropical deforestation is an important process that contributes to the present-day concern for the loss of biodiversity and increased rates of species extinction (Didham et al. 1996). With the expansion and consolidation of agricultural frontiers, species that survive in habitat remnants are confronted with a modified environment immersed in environmentally harsh matrices, such as pastures and croplands (Gascon et al. 2000, Tabarelli et al. 2004). Such changes directly influence the number of species and size of populations by affecting biotic interactions, the amount of available resources and the extent of variations in the physical environment (Schiffler 2003).

In northeastern Brazil, between 30.4% and 51.7% of the semi-arid Caatinga ecosystem has been altered by human activities (Leal et al. 2005). Inappropriate land use has caused serious environmental harm and the accelerating process of desertification is currently threatening about 15% of the Caatinga region (Leal et al. 2005). Moreover, the rich, diversified biota of Caatinga is poorly protected, with only 11 reserves (less than 1% of the region) set aside as strictly protected areas (Leal et al. 2005). With growing pressure of human development on natural resources, identifying and making use of ecological indicators is an essential task in the conservation of tropical ecosystems (Uehara-Prado et al. 2009).

Dung beetles play an important role in tropical ecosystems with regard to the recycling of soil nutrients (Bornemissza and Williams 1970) and secondary seed dispersal (Estrada and Estrada 1991), thereby contributing to the maintenance of soil fertility and natural processes of environmental regeneration (Avendaño-Mendoza et al. 2005, Quintero and Roslin 2005). These beetles have been widely proposed as an ideal group for biodiversity inventories and monitoring, as they satisfy all of the criteria of a focal taxon and have been used in ecological research, biodiversity surveys and conservation work in many regions of the world (Spector 2006). Dung beetles are found in defined guilds that are functionally and taxonomically dependent upon the environment. Such guilds are established according to the strategy used for allocating feeding or nesting resources (telecoprids, paracoprids and endocoprids), the degree of dietary specialization (coprophagous, necrophagous, saprophagous, mycetophagous, myrmecophilous, predatory or generalist) and the temporal pattern of their activity (diurnal, nocturnal, twilight or continuous) (Halffter and Matthews 1966, Cambefort and Hanski 1991, Halffter and Favila 1993, Hernández 2002).

A number of studies carried out in the neotropics have demonstrated that dung beetle communities are susceptible to habitat changes (Nichols et al. 2007, Gardner et al 2008, Larsen et al. 2008, Hérnandez and Vazde-Mello 2009, Neves et al. 2010, Silva et al. 2010, Filgueiras et al. 2011). Micro-environmental variations in soil texture, humidity and forest structure affect the occurrence and relative abundance of these communities (Spector and Ayzama 2003). The destruction of the natural environment for the conversion of such areas into pastures or monocultures alters local abiotic factors, which affect both the population structure and composition of the beetle community (Medri and Lopes 2001). These changes decrease evenness in the community, leading to the dominance of some species, as most interior forest species cannot tolerate the conditions offered by deforested areas (Davis et al. 2001, Escobar 2000). Such patterns have mainly been observed in tropical rainforests. However, studies involving dung beetles are scarce (Andresen 2005, Hernández 2005, 2007, Lopes et al. 2006, Liberal 2008). These beetles are also affected by seasonal changes in rainfall (Hanski and Cambefort 1991), with differences in abundance and guild structure between the dry and wet seasons (Andresen 2005).

The aim of the present study was to compare dung beetle communities in a land use area and an undisturbed area in the Caatinga and answer two questions: (1) how does forest disturbance and rainfall affect dung beetle communities in their abundance, species richness, and diversity? (2) Does disturbance change dung beetle community structure?

## Materials and Methods

### Study site

**Figure 1.**
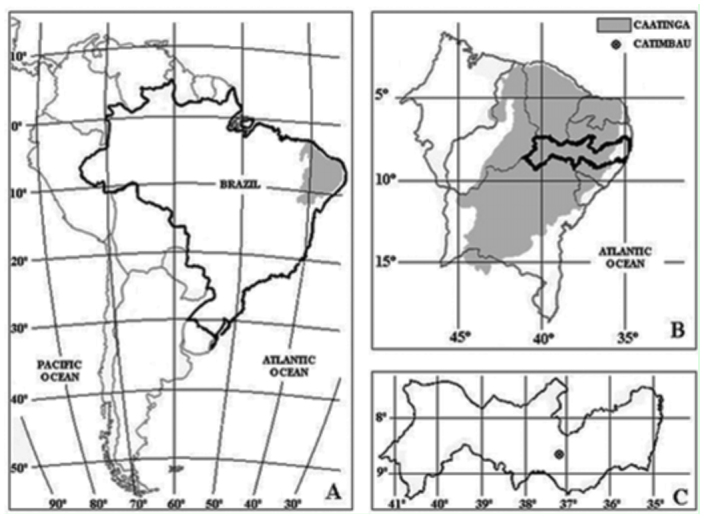
Location of the study area, Caatinga area of Catimbau National Park, Northeastern Brazil. (A) South America, (B) Northeastern Brazil, (C) State of Pernambuco. High quality figures are available online

A study was carried out in Catimbau National Park (08° 37′ S, 37° 09′ W), located in the state of Pernambuco, northeastern Brazil (Figure 1). The area is part of the *Sertaneja* depression, corresponding to the largest geomorphological feature of the semiarid region, which consists of a large depressed plain bordered by elevated areas and mountain ranges (Andrade-Lima 1981). This Brazilian ecosystem is known as the Caatinga and consists of patches of seasonally dry forest and sclerophyllous vegetation (*sensu* Pennington et al. 2000), covering 730,000 km^2^ of the semiarid region in Brazil (Sampaio 1995). Since 2002, Catimbau National Park has been a Federal Conservation Unit. This designation was given to preserve the geological, speleological and archeological features as well as to protect one of the last preserved areas of Caatinga. The climate is defined as hot semiarid (BSh—according to the Köppen climate classification), with an annual mean temperature of 25° C and rainfall ranging from 700 to 1100 mm year^-1^ (Andrade-Lima 1981; ITEP 2006). The temperature can reach as high as 45° C in the hottest period. Rainfall occurs between March and July (ITEP 2006). In the present study, months with rainfall indices greater than 60 mm were considered the wet season and the remaining months were considered the dry season. These data were provided by the Brazilian Geological Service.

### Methodology

Dung beetle communities were sampled monthly from May 2006 to April 2007 (except February 2007) in two environments denominated “land use area” and “undisturbed area.” The land use area was used for raising cattle in the dry season and corn farming in the wet season. The undisturbed area is a preserved area in which there has been no constant tree cutting or livestock raising.

Three areas spaced 400 m apart were chosen in each environment. Pitfall traps consisted of a plastic container (9.5 cm in diameter and 20 cm in depth) and human feces were used as bait. The traps were buried in the soil at the end of the day, protected by a suspended polystyrene disk and remained exposed for 48 h. Six traps were deployed in each area, distributed randomly but at a minimal distance of 15 m from each other. A total of 396 samples were collected (six traps · three sampling points · two habitats · 11 sampling periods). The insects collected were incorporated into the Entomology Collection of the Universidade Federal de Pernambuco (Brazil).

### Data analysis

The dung beetle communities were studied with respect to abundance, species richness, equitability (Camargo index) and diversity [Shannon-Wiener diversity index (Log_2_)]. Abundance and species richness were compared between the two environments (land use area and undisturbed area) and seasons (dry and wet) using generalized linear models with a Poisson error distribution. The dung beetle communities were studied using abundance, species richness, equitability (Camargo index), and diversity data [Shannon-Wiener diversity index (Log_2_)]. Camargo's index (E ') is not affected by species richness and is used when you want rare and common species have equivalent weight in the sample. Diversity was compared between the two environments (land use area and undisturbed area) and seasons (dry and wet season) using the Two-Way ANOVA test. The estimation of these indices was performed using the Ecological Methodology 5.2. program (Kenney and Krebs 2000) and analyses were performed with the STATISTICA 6.0 program (STATSOFT 1998), with the significance level set at 0.05. Rarefaction curves for the land use area and undisturbed area were constructed using individual-based rarefaction (Gotelli and Colwell 2001). Non-metric multidimensional scaling was performed on the resemblance matrix of Bray-Curtis distances, with 1000 random restarts, to determine whether disturbance affected species composition (presence/absence species data) and the abundance of the dung beetle communities. The species abundance data were transformed (square root) and standardized (sensu Clarke and Gorley, 2001) in order to avoid any bias resulting from highly abundant species. Analysis of similarities (Clarke, 1993) was performed on the matrix of Bray-Curtis similarities, with 999 permutations, to test the null hypothesis of equal species composition between the land use area and undisturbed area. Finally, Spearman's correlation coefficient was used to determine whether rainfall was correlated with abundance and species richness.

## Results

A total of 1097 dung beetles were captured at all collection sites, representing 13 species, six genera and four tribes. In the undisturbed area, 371 individuals representing five species were found. In the land use area, 726 individuals representing 11 species were found. No differences in the diversity index or Camargo equitability index were found between the two environments (F = 0.36, df = 1, *p* = 0.56); (F = 0.78, df = 1, *p* = 0.39).

A total of 1021 individuals representing 13 species were caught in the wet season and 76 individuals representing six species were caught in the dry season. Significant differences were found in abundance (F = 363.02, df = 1, *p* < 0.0001) between seasons. Moreover, a significant difference was found in diversity index values between seasons (F = 29.59, df = 1, p < 0.0001), whereas no statistically significant seasonal differences were found with regard to species richness.

**Figure 2.**
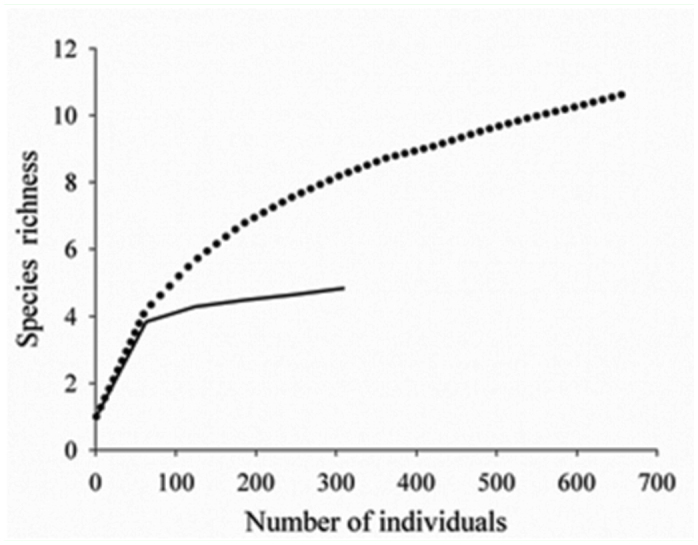
Individual-based rarefaction curves for dung beetles in land use area (dots) and undisturbed area (line), in two environments of Catimbau National Park, North. High quality figures are available online

Significant differences in abundance (F = 47.0, df = 5, *p* < 0.0001) were found between environments, with higher values in the land use area. However, even though the land use area exhibited higher species richness than the undisturbed area (Figure 2), the generalized linear model did not identify distinction in terms of dung beetle species richness.

Only *Dichotomius geminatus* Arrow (Coleoptera: Scarabaeidae) occurred in every sample and was the most abundant species found at both sites (74.93%). *Ateuchus* aff. *carbonarius* Harold was the second most abundant species in the land use area and *Dichotomius* aff. *laevicollis* Felsche was the second most abundant species in the undisturbed area. *Dichotomius* aff. *laevicollis* was restricted to the undisturbed area (Table 1). Only 23.08% of the species were rollers and 76.92% were paracoprids.

**Figure 3.**
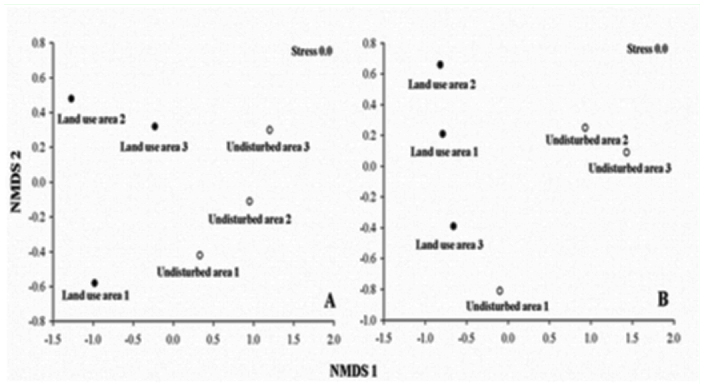
Nonmetric multidimensional scaling (NMDS) ordination of land use and undisturbed area, based on dung beetle communities. NMDS (A) shows the difference in terms of species composition (presence/absence species data) and NMDS (B) shows the difference based on number of individuals. High quality figures are available online

The non-metric multidimensional scaling results for the dung beetle communities clearly revealed different species composition and community structure (abundance data) between the land use area and the undisturbed area, with the exception of one undisturbed area (Figure 3). These results were confirmed by analysis of similarities (global R = 0.81; *p*<0.01) (presence/absence species data) and (global R = 0.70; *p*< 0.01) (abundance data).

**Figure 4.**
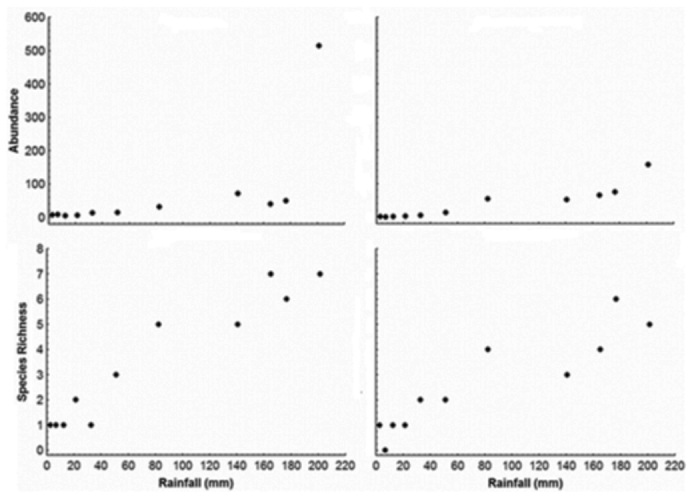
Correlation between dung beetle abundance and species richness of Scarabaeinae with rainfall in two environments of Catimbau National Park, North. High quality figures are available online

**Table 1.**
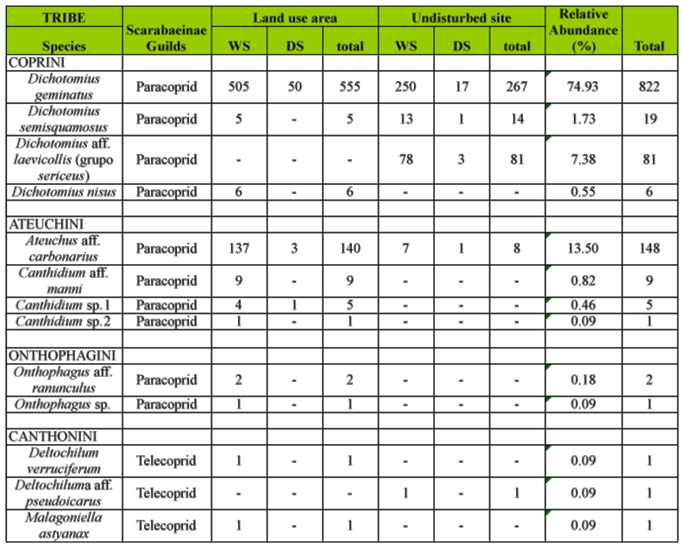
Scarabaeinae gilds, number of specimens, and relative abundance (%) of Scarabaeinae species present in two environments of Catimbau National Park, Northeastern Brazil. WS: wet season, DS: dry season.

An increase in both abundance and species richness was found at the beginning of the wet season. Spearman's correlation coefficients were high and significant for abundance (land use area: R = 0.63, N= 11, *p* = 0.0377; undisturbed area: R = 0.91, N = 11, *p* = 0.0001) and species richness (land use area: R = 0.96, N = 11, *p* = 0.0002; undisturbed area: R = 0.92, N = 11, *p* = 0.0007) (Figure 4), both of which were positively correlated with rainfall.

## Discussion

The results of the present study demonstrate the impact of human activities on dung beetle communities in a semi-arid ecosystem in Brazil. However, it was documented that the communities in the undisturbed area were impoverished in comparison to those in the land use area. The communities also proved to be influenced by rainfall.

The number of individuals nearly doubled in the land use area. The greater abundance in this environment was mainly due to *Dichotomius geminatus,* a species that is well—distributed throughout the Caatinga (Hernandez 2005, 2007; Lopes et. al, 2006) and well-adapted to pasture areas (e.g. Schiffler et al. 2003). Low equitability in the community structure resulting from the domination of a single species may be associated with habitat disturbances, such as vegetation cutting or livestock grazing (Davis et al. 2001; Halffter and Arellano 2002). Due to environmental alterations, the dung beetle community may have suffered from changes to its original structure (Halffter and Favila 1993), with a considerable reduction in diversity. Species adapted to the new conditions tend to invade the newly modified environment and a local increase in species richness may be observed (Halffter and Favila 1993; Davis et al. 2001).

Species richness may have been influenced by human impact, despite the low abundance of most species. When grazing pressure is excessive, diversity drops due to mortality in species that are less resistant to this type of disturbance. Adaptations that enhance survival in semi-arid environments may promote grazing tolerance or avoidance because water stress and grazing stress are similar in the sense that both result in a loss of vegetation cover (Verdú et al. 2007). Therefore, in dry environments, resource availability becomes an important factor to both the abundance of individuals and species richness (Hanski and Cambefort 1991). The greater richness found at disturbed sites in Catimbau National Park (rarefaction curves) may be related to the continuous supply of resources, as these areas were close to cattle raising sites.

The presence of *Ateuchus* sp. *D. geminatus,* and *D. semisquamosus* Curtis in one undisturbed area resulted in the formation of a consistent cluster with land use areas (see Figure 4). This finding may be explained by the fact that these species were also found in land use areas. The presence of some species in certain localities may indicate the quality of the environment (Halffter and Favila 1993). For instance, *Dichotomius nisus* Olivier was only found in the land use area; Schiffler et al. (2003) and Vieira et al. (2008) describe a similar finding in pasture areas. *Ateuchus* aff. *carbonarius* was significantly more common in the land use area; this species is common in pastures in other areas of the Caatinga (Hernandez 2007). *Dichotomius* aff. *laevicollis* (group *sericeus*) occurred only in the undisturbed area. Schiffler et al. (2003) found this species in great abundance in an alluvial forest, but not in pasture areas and Vieira et al. (2008) report this species in the original vegetation of restinga forest. Thus, *Dichotomius* aff. *laevicollis* may be considered a potential indicator of undisturbed areas.

The tribe Canthonini, often found in the Caatinga (Hernández 2005), is commonly represented by *Canthon.* However, there were no individuals from this genus in the present study. The fact that the pitfall traps were deployed at the end of the day may have prevented the capture of individuals with diurnal behavior. While activity periodicity was not evaluated in the present study, the dung beetle species represented here were mostly paracoprids, with only three telecoprids. Paracoprids species are generally more active at dawn (Krell-Westerwalbesloh et al. 2004). These beetles avoid high diurnal temperatures, which may elevate the body temperature to lethal levels, especially in bigger and darker species. Such is the case with the genus *Dichotomius,* which was the most abundant in the present study. Moreover, the dark coloration of nocturnal species may have evolved as an adaptation in order to avoid predation (Hernández 2002). On the other hand, telecoprid species are frequently diurnal; the increase in body temperature increases the mobility of these beetles, thereby favoring the construction of excrement balls (Krell-Westerwalbesloh et al. 2004, Halffter et al. 2008).

The highest abundance values were found at the beginning of the wet season. This pattern has also been reported in other areas of the *Caatinga* (Hernández 2005, 2007) as well as in semiarid environments in Mexico (Andresen 2005) and some areas of South Africa (Davis 1996). Many dung beetle species experience a peak of emerging adults at the beginning of the rainy period (Cambefort and Hanski 1991, Andresen 2005), particularly in dry tropical regions, which are highly influenced by rainfall (Halffter and Mathews 1966, Hanski and Cambefort 1991).

The notable variations in abundance and diversity of dung beetle communities in semiarid environments may be due to the extreme conditions that occur in dry periods. The dry season in the Caatinga is characterized by a reduction or complete absence of rainfall, along with high solar incidence and low humidity (Reis 1976). These conditions lead to dehydration and, consequently, a reduction in excrement availability, an emphemeral resource. Prolonged periods of low primary production also hamper the maintenance of populations of large mammals in the Caatinga (Hernández 2005), which are the main producers of the manure used by dung beetles for feeding and nesting (Halffter and Mathews 1966).

In conclusion, while some findings revealed differences in the structure of the dung beetle communities between the different environments sampled, it is not possible to declare that this is a pattern for the Caatinga. The low degree of species richness may have hindered the understanding of the influence of changes on these communities, except in an isolated fashion for a few species. On the other hand, seasonal variation proved to be an important factor determining dung beetle diversity, abundance and species richness. Further studies should examine the impact of human activities on dung beetle communities in the Caatinga and determine the validity of the causal mechanisms proposed.
